# Shifting effects of host physiological condition following pathogen establishment

**DOI:** 10.1098/rsbl.2022.0574

**Published:** 2023-03-01

**Authors:** Kate E. Langwig, A. Marm Kilpatrick, Macy J. Kailing, Nichole A. Laggan, J. Paul White, Heather M. Kaarakka, Jennifer A. Redell, John E. DePue, Katy L. Parise, Jeffrey T. Foster, Joseph R. Hoyt

**Affiliations:** ^1^ Department of Biological Sciences, Virginia Polytechnic Institute, Blacksburg, VA 24061, USA; ^2^ Department of Ecology and Evolutionary Biology, University of California, Santa Cruz, CA 95060, USA; ^3^ Wisconsin Department of Natural Resources, Madison, WI 53707, USA; ^4^ Michigan Department of Natural Resources, Baraga, MI 49908, USA; ^5^ Pathogen and Microbiome Institute, Northern Arizona University, Flagstaff, AZ 86011, USA

**Keywords:** body mass, emerging infectious disease, wildlife disease, population impacts, white-nose syndrome, *Pseudogymnoascus destructans*

## Abstract

Understanding host persistence with emerging pathogens is essential for conserving populations. Hosts may initially survive pathogen invasions through pre-adaptive mechanisms. However, whether pre-adaptive traits are directionally selected to increase in frequency depends on the heritability and environmental dependence of the trait and the costs of trait maintenance. Body condition is likely an important pre-adaptive mechanism aiding in host survival, although can be seasonally variable in wildlife hosts. We used data collected over 7 years on bat body mass, infection and survival to determine the role of host body condition during the invasion and establishment of the emerging disease, white-nose syndrome. We found that when the pathogen first invaded, bats with higher body mass were more likely to survive, but this effect dissipated following the initial epizootic. We also found that heavier bats lost more weight overwinter, but fat loss depended on infection severity. Lastly, we found mixed support that bat mass increased in the population after pathogen arrival; high annual plasticity in individual bat masses may have reduced the potential for directional selection. Overall, our results suggest that some factors that contribute to host survival during pathogen invasion may diminish over time and are potentially replaced by other host adaptations.

## 1. Introduction

The introduction of novel pathogens to naive hosts can have profound effects on populations [1–7]. Hosts may survive initial pathogen invasion through multiple mechanisms including evading infection or pre-adaptive traits that allow for survival despite infection or disease [8,9]. Generally, pre-adaptive traits that confer a survival advantage might be directionally selected to increase in frequency in the population in the presence of a pathogen. However, if pre-adaptive traits have strong trade-offs, or are highly plastic (e.g. environmentally dependent), factors enabling hosts to survive during initial pathogen invasion may not confer any advantage subsequently [10,11]. Ultimately, traits that determine long-term host–pathogen coexistence may take longer to evolve and become widespread than traits allowing for initial survival, particularly if such traits provide stronger protection than pre-adaptive mechanisms [10,12–14].

Factors that affect the probability of host survival with invasive pathogens include, but are not limited to, age, chronic disease, prior exposure and body mass [15]. In general, hosts with adequate fat stores, high nutrient levels, and access to high-quality habitat should demonstrate improved survival with disease over weaker hosts. However, within individuals, host body condition can be highly variable across seasons and years leading to heterogeneity in the relationship between host body condition and disease, and possibly make it a less reliable mechanism for long-term survival [16]. Variable effects of body condition may be particularly pronounced when there is highly seasonal availability of food sources, leading to high stochasticity among individuals in their ability to consistently maintain high body condition when faced with annual disease outbreaks.

White-nose syndrome (WNS) is a seasonal annual epizootic of bats caused by the fungal pathogen *Pseudogymnoascus destructans* [17–20]. WNS was first detected in New York, USA in 2006, and has caused widespread declines in hibernating bat populations across North America [6,21,22]. *Pseudogymnoascus destructans* grows optimally in cool conditions (1–17°C) [23], resulting in annual winter epidemics that occur when bats begin hibernating [18]. Invasion of *P. destructans* into bat skin tissue causes severe physiological disruption, elevating bat metabolic rate and increasing respiratory acidosis [24,25]. Bats, in turn, arouse to normalize blood pH, which further increases evaporative water loss and causes dehydration. Higher energy expenditure from infection increases fat loss and emaciation, which frequently leads to mortality [26–28].

Increases in stored fat and improved budgeting of fat overwinter are therefore hypothesized to be important mechanisms determining bat survival with WNS [29–31], which typically increases within 4–5 years of WNS arrival after initially severe declines [6,17,32,33]. However, other mechanisms of host persistence have also been described, including potential increases in host resistance through immunity or microbially mediated reductions in pathogen growth [17,33], and movement toward colder roosting conditions, which limits fungal replication [34,35]. Nonetheless, changes in body mass have the potential to have strong effects on bat survival, but comprehensive analyses of the effect of body mass on individual bat survival with WNS in the field have yet to be conducted. In addition, because host body condition may exhibit high annual variability [36], the importance of body mass as a sustained factor affecting population persistence with WNS merits additional investigation. Here, we investigate changes in the effect of body mass on the survival of individual little brown bats (*Myotis lucifugus*) during the invasion and establishment of *P. destructans* across 24 sites in eastern and central USA during winter hibernation. We hypothesized that fat might be an important pre-adaptive mechanism enabling bats to survive during pathogen invasion and aimed to test whether fat retained its importance as might occur due to directional selection or whether the positive effects of high body mass diminished over time as disease resistance increased in populations.

## 2. Methods

We studied the arrival and establishment of *P. destructans* at 24 hibernacula (caves and mines where bats spend the winter) in Virginia, Wisconsin, Illinois and Michigan over 7 years (electronic supplementary material, tables S1–S3) [37]. We visited sites twice per winter and collected data on infection status and body mass of bats. At each site, we sampled individual bats (electronic supplementary material, table S1, mean = 9.2, range: 1–50) stratified across site sections. Because sites used in this study were primarily small mines where it was possible to observe all bats present, in many instances, all individuals in the population were sampled. For each bat, we collected a standardized epidermal swab sample [18], attached a unique aluminium band and measured body mass using a digital scale (GDealer, accuracy ± 0.03 g). Because common condition indices are no more effective than body mass for estimating fat stores [38], we did not include information on bat forearm size in order to reduce handling disturbance. At every visit, we recorded and resampled any previously banded bats present. We stored swabs in RNAlater, and samples were kept at 0°C while in the field, and then at −20°C until processing. We tested samples for *P. destructans* DNA using real-time PCR and quantified fungal loads [21,39]. Animal handling protocols were approved by Virginia Tech IACUC (no. 17-180, no. 20-150).

We investigated the effect of bat early hibernation (November) body mass on the probability an individual was recaptured overwinter (e.g. within-winter) using a generalized linear mixed model with a binomial distribution and a probit link, with site as a random effect, and body mass and disease phase (epidemic = 1–3 years since pathogen arrival, or established = 4–7 years since pathogen arrival) as interacting fixed effects (electronic supplementary material, table S1, total *N* individuals = 775). Phases were established based on previous results demonstrating that populations approach stability by year 4 following WNS arrival [6,32] For analyses of individual survival and body mass, results were similar whether we used categorical disease phase or years since WNS as a continuous variable (electronic supplementary material, appendix) and grouping by phase maximized the number of bats in the epidemic years when mortality was high and the number of recaptured bats was low. For bats that were recaptured overwinter, we examined the effect of early winter body mass and infection on the amount of mass lost overwinter during both the epidemic and established phase using a linear mixed model with site as a random effect and the change in body mass as the response variable and fixed effects of early winter mass interacting with early winter fungal loads with additional additive effect of disease phase (electronic supplementary material, table S2, total *N* = 158). Finally, we explored changes in mass over time since the invasion of *P. destructans* on an individual and population level to examine both plasticity and phenotypic change. For bats that were recaptured in multiple years, we used a linear mixed model with mass as a response variable, years since WNS as a fixed effect and bat band ID as a random effect to explore plasticity in whether individual bat mass changed over time (*N* = 91 observations, 42 unique bands, 1–3 recapture events per bat, electronic supplementary material, appendix 4.0.3). At a population level, bat declines in sites with the best invasion mass data limited our ability to explore changes in mass, so we restricted our analyses to *N* = 5 sites (electronic supplementary material, table S3) that were measured during invasion and had sufficient bats to estimate during established periods using log_10_ mass as our response variable (logged to normalize) and years since WNS interacting with season with site as a random effect. All analyses were conducted in R v.4.1.2 using lme4 [40].

## 3. Results

**Figure 1.**
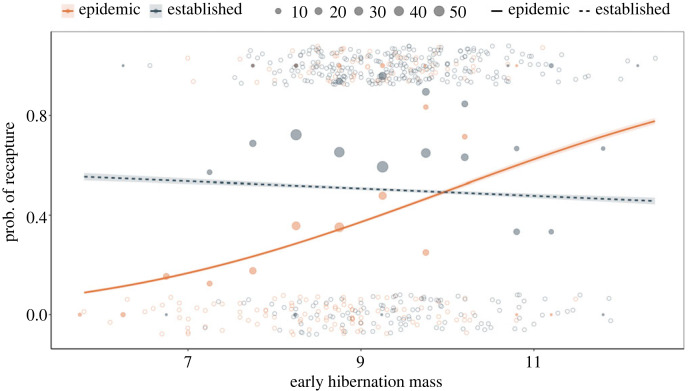
The effects of body mass during early hibernation on the probability of little brown bat recapture vary with time since *P. destructans* arrival. In years 0–3 post *P. destructans* arrival, the probability a bat was recaptured overwinter increased as early hibernation mass increased. However, after WNS established (greater than 3 years since *P. destructans* arrival), there was no longer a clear trend between early hibernation body mass and bat survival. Solid points of early hibernation body masses during each phase show the fraction recaptured at 0.5 g bins (e.g. 9.75–10.25) and sample sizes for binned data. Shaded regions show 95% confidence intervals.

As WNS invaded and caused massive declines in bat populations, bats that were heavier in early winter were more likely to be recaptured than lighter ones (figure 1; slope of mass versus recapture during invasion ± s.e.: 0.320 ± 0.14, *p* = 0.0220). However, after WNS established in sites (years 4–7 following *P. destructans* detection), recapture overall was higher than during the epidemic (invasion versus establishment coef: 3.551 ± 1.46, *p* = 0.0152), and the effect of mass on the probability of recapture was significantly lower than the epidemic phase (interaction slope: −0.357 ± 0.16, *p* = 0.0250), and the slope of mass versus recapture did not differ significantly from 0 (electronic supplementary material, appendix 1.0.3).

**Figure 2.**
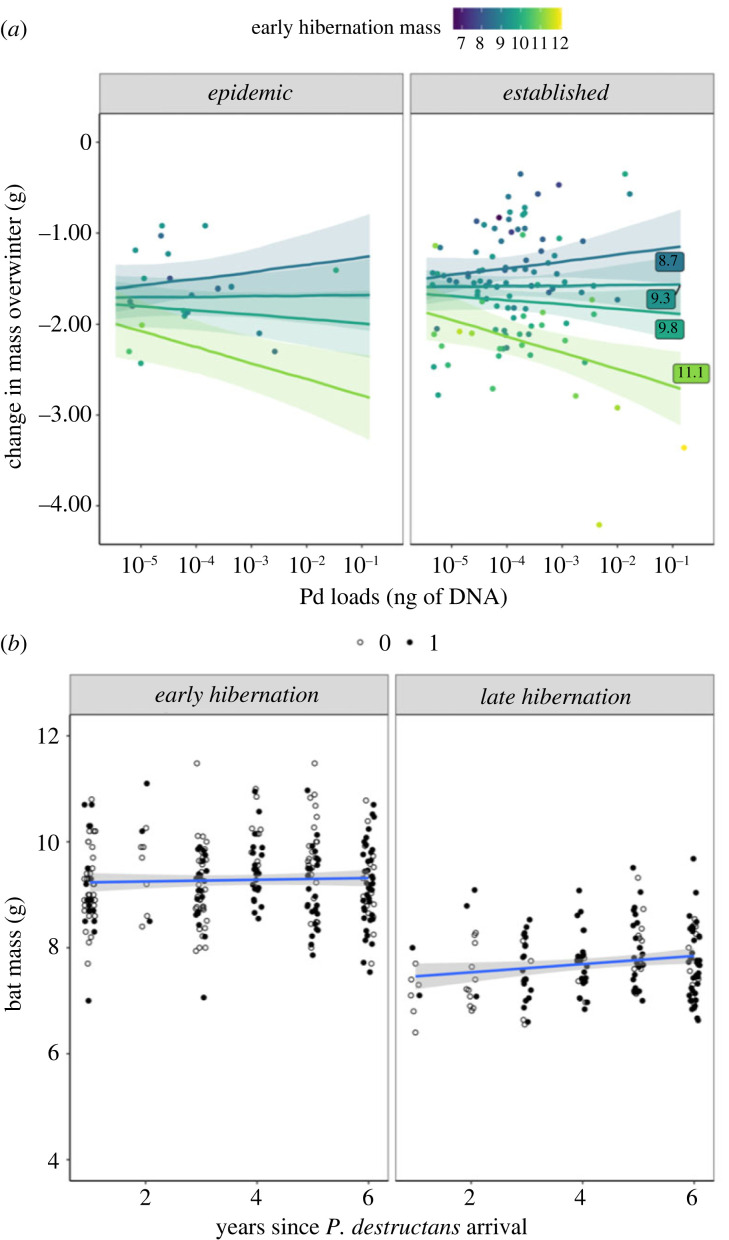
(*a*) Fungal loads and early hibernation (November) body mass of little brown bats strongly influences the change in individual bat mass over winter. Points show individual bats captured in both early and late hibernation. Colours denote masses of bats during early hibernation and labelled lines show predictions based on the 25th (8.7 g), 50th (9.3 g), 85th (9.8 g) and 95th (11.1 g) percentiles of the early hibernation masses. Shaded regions show 95% confidence intervals. Bats that have higher initial body mass lost more weight over winter than bats with lower body mass (i.e. darker lines are higher), suggesting that bats expend fat stores accordingly over winter. In addition, fungal loads significantly modify the effect of early hibernation mass on mass lost overwinter. Bats with high infections that were heavier lose more mass than similarly infected bats that were lighter, suggesting that highly infected bats that survive to be recaptured expend fat in accordance with their infection status. (*b*) Average body mass of banded little brown bats in early (November) and late (March) hibernation that were recaptured (filled circles) or not recaptured (open circles) overwinter during the WNS epidemic (years 0–3) and WNS established period (years 4+) at the same sites over time (*N* = 5). Shaded regions show 95% confidence intervals. We found no clear support that hibernation body masses of bats increased over time when examining these data continuously (top) but marginal support categorically (electronic supplementary material, figure S1).

For bats that survived overwinter and were recaptured, mass lost overwinter depended on both early hibernation weight and infection, and their interaction (figure 2*a*; electronic supplementary material, appendix 2.02, early winter mass : early winter infection interaction coef: −0.112 ± 0.04, *t* = −2.742, *p* = 0.0095). There was little support for including disease phase as a predictor (established coef: 0.396 ± 0.21, *t* = 1.940, *p* = 0.270), likely due to the paucity of bats recaptured during the epidemic phase when mortality was high (electronic supplementary material, table S2, *N* = 30 bats recaptured during epidemic phase). Generally, bats that were heavier lost more weight overwinter than bats that were lighter (coef: −0.737 ± 0.16, *t* = −4.613). However, the degree of mass lost depended strongly on an interaction with fungal loads such that bats appeared to expend fat in accordance with both infection and stored fat. Bats that had high body mass in early hibernation and were highly infected lost the most mass overwinter. However, lighter bats with equivalently severe infections lost less weight (electronic supplementary material, appendix 2.0.2).

We found limited support for increases in mass at a population level. Including years since pathogen arrival as a continuous effect, we found no clear support for increases in mass at a population level (years since pathogen invasion coef: 0.002 ± 0.01, *t* = 0.983, figure 2*b*, electronic supplementary material, table S3 and appendix 3.0.2). We did find support for a modest increase in log_10_ early hibernation body mass between the epidemic and established periods at five sites that were sampled at all time points in most years (established coef: 0.011 ± 0.01, *t* = 2.031, electronic supplementary material, figure S1 and appendix 3.0.4); however, this was largely due to an increase between one annual time step (year 3 to year 4). We found no support for an increase in mass due to plasticity (electronic supplementary material, appendix 4). Using just recaptured bats, we found weak and unclear support for increases in log_10_ early hibernation body mass with years since WNS establishment (0.004 ± 0.003, *t* = 1.508, electronic supplementary material, figure S1 closed circles, electronic supplementary material, table S2 and appendix 4.0.2). Furthermore, masses of individual bats that were recaptured in multiple years decreased non-significantly (−0.002 ± 0.003, *t* = −0.701, electronic supplementary material, figure S2 and appendix 4.03). Among individual bats recaptured annually, there was high plasticity in body mass which ranged from −1.78: +1.09 g, suggesting that bat fat stores may be highly dependent on local conditions in summer and autumn.

## 4. Discussion

We found that the relationship between body mass and survival waned as the epidemic progressed. Furthermore, fat loss in bats increased with initial stored fat, as has been previously found in another species [41], and was significantly modified by infection, suggesting that highly infected light bats use fat more conservatively than heavier bats, indicating that bats surviving with disease may budget fat stores to mitigate the physiological disruption posed by WNS. Importantly, we did not find evidence that bat survival once the disease established was enhanced by increases in the amount of stored fat [29]. We found mixed support that fat increased at the population level as the disease established. When treating years since pathogen arrival continuously, there was no clear trend of increases in fat at the population level. In some years, annual increases in fat occurred, but these were modest relative to the range of body conditions at the start of hibernation (recaptured bats during the established WNS period ranged from 7 to 12 g and gains were an average of 0.18 g). We also found no support of consistent mass increases in individual bats, and year to year fat stores were highly variable (range −1.78: +1.09 g).

There are several potential reasons that could explain why the importance of fat changed as *P. destructans* established. First, the initial epizootic may have selected for fatter individuals, thus making the effects of fat less apparent as the pathogen established. However, body mass differences between the invasion and established phases were very modest relative to annual plasticity in bat masses and support for increased mass at the population level was mixed. Second, demographic changes in bat populations could result in sex or age-based cohorts (and thus the effect of mass on survival) changing as WNS established. While we did not routinely collect age data due to the difficulties of ageing little brown bats more than six months after birth, other studies have indicated no changes in the number of juveniles in the summer maternity season [42]. Sex-biases in infection have reduced the number of female bats as WNS established [43]; however, females are generally slighter heavier than males which should result in a pattern opposite of our findings. Third, bats in some populations have evolved higher pathogen resistance [33,35] which may have reduced selection for increased body mass, particularly if fatter bats face other trade-offs, such as reduced flight abilities [44,45]. Fourth, bats have shifted to using cooler microclimates that also reduce the growth of the fungus, resulting in less severe disease [34]. Fifth, changes in the pathogen (e.g. a reduction in virulence) could have enabled more hosts to survive, thus experiencing fewer adverse effects (e.g. excess fat loss) from the pathogen [46,47]. Lastly, bats may have adapted to the physiological disruption posed by infection, as evidenced by the relationship between mass loss, infection and early hibernation weight. This finding is consistent with the hibernation optimization hypothesis [48–50], suggesting that bats do not use a fixed amount of fat during hibernation [51,52], and generally aligns with findings conducted on unaffected little brown bats that demonstrated increases in arousals with increases in early hibernation fat [48]. Overall, increased fat stores may have been beneficial initially, but changes in other host or pathogen traits may have relaxed selection on fat over time.

Our results have important implications for the conservation of bats impacted by WNS. Supplemental feeding and enhancement of autumn bat habitat to increase insect prey abundance have been explored as management strategies to increase bat fat stores to reduce WNS impacts [53]. Our results suggest that this could be effective prior to or during pathogen invasion. However, it may provide less benefit to bats once the pathogen has established for several years. We find that bats expend fat in accordance with their infection severity and initial fat stores, suggesting that supplemental feeding might not achieve the desired benefit of enhancing bat survival if bats simply alter fat use accordingly. In addition, supplemental feeding of wildlife may have unexpected negative consequences, including increases in predation, increases in susceptibility due to less nutritious food sources, and enhancement of pathogen spread due to host aggregation [54], and these potential negative effects should be carefully considered before wide-scale implementation.

Species survival in the face of global change will likely require rapid adaptation and change itself may outpace the speed at which species can evolve [55,56]. For species and populations that persist, some traits that may be beneficial for initial survival may prove less important over time [9,57]. This phenomenon may be partly explained by coevolutionary theory, which suggests that both hosts and pathogens must constantly adapt and innovate in order to maintain high fitness [12]. Ultimately, developing a more comprehensive understanding of the pre-adaptive factors that aid in population health can enable us to build more resilient populations in the Anthropocene.

## Data Availability

The datasets generated in this study are available from the Dryad Digital Repository: https://doi.org/10.5061/dryad.wh70rxwrv [37] including a metadata file. Exact site locations are not disclosed to protect endangered species and landowners. Additional data summaries are provided in the electronic supplementary material [58].
